# Illuminating the Tiny World: A Navigation Guide for Proper Raman Studies on Microorganisms

**DOI:** 10.3390/molecules29051077

**Published:** 2024-02-29

**Authors:** Sandra Baaba Frempong, Markus Salbreiter, Sara Mostafapour, Aikaterini Pistiki, Thomas W. Bocklitz, Petra Rösch, Jürgen Popp

**Affiliations:** 1 Institute of Physical Chemistry and Abbe Center of Photonics, Friedrich Schiller University, Helmholtzweg 4, 07743 Jena, Germany; sandra.frempong@uni-jena.de (S.B.F.); markus.salbreiter@uni-jena.de (M.S.); sara.mostafapourghasrodashti@uni-jena.de (S.M.); aikaterini.pistiki@uni-jena.de (A.P.); thomas.bocklitz@uni-jena.de (T.W.B.); juergen.popp@uni-jena.de (J.P.); 2 InfectoGnostics Research Campus Jena, Center of Applied Research, Philosophenweg 7, 07743 Jena, Germany; 3 Leibniz-Institute of Photonic Technology, Member of the Leibniz Research Alliance-Leibniz Health Technologies, Albert-Einstein-Str. 9, 07745 Jena, Germany; 4 Cluster of Excellence Balance of the Microverse, Friedrich Schiller University Jena, 07743 Jena, Germany

**Keywords:** Raman spectroscopy, bacteria, single-cell analysis, sample isolation, machine learning

## Abstract

Raman spectroscopy is an emerging method for the identification of bacteria. Nevertheless, a lot of different parameters need to be considered to establish a reliable database capable of identifying real-world samples such as medical or environmental probes. In this review, the establishment of such reliable databases with the proper design in microbiological Raman studies is demonstrated, shining a light into all the parts that require attention. Aspects such as the strain selection, sample preparation and isolation requirements, the phenotypic influence, measurement strategies, as well as the statistical approaches for discrimination of bacteria, are presented. Furthermore, the influence of these aspects on spectra quality, result accuracy, and read-out are discussed. The aim of this review is to serve as a guide for the design of microbiological Raman studies that can support the establishment of this method in different fields.

## 1. Introduction

The identification of bacteria or other microorganisms is commonly based on culture-based techniques. These techniques, however, are not always applicable in a straightforward manner, with issues appearing in many cases. Medical samples like, e.g., blood, contain only a limited amount of bacteria/mL, thus requiring enrichment methods like blood cultures. Other medical samples may include contamination from the body flora which has to be separated from the causative pathogen. In these cases, pure cultures are required, that need to be obtained with repeated and time-consuming sub-culturing. All in all, such cultivation-based isolation techniques require from 12 h up to several days before the causative pathogen may be identified [1]. For environmental samples this step may be even more challenging since many bacteria are hardly or even not cultivable [2].

In recent years, the importance of Raman spectroscopy for the characterization and identification of bacteria has increased. On the one hand, Raman spectroscopy, as a vibrational spectroscopic technique, enables the identification of bacteria using their optical fingerprint [3,4,5,6,7,8,9]. On the other hand, Raman spectroscopy can be used to monitor chemical changes in bacterial cells [10,11,12,13]. 

**Figure 1:**
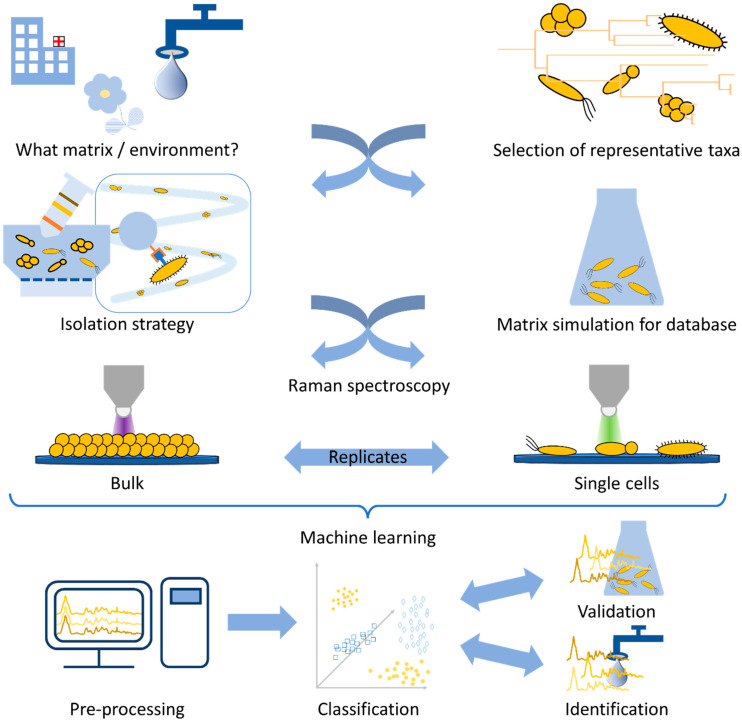
Schematic workflow for a Raman spectroscopic study on microorganisms.

In this review, we demonstrate the workflow for establishing a reliable Raman database. As can be seen in Figure 1, the first question arising is always what microorganisms can be found in the chosen environment. For the database to be established, it is important to choose the representative taxa in such a way that a variety of strains/species/genera are included which can be found in the habitat under investigation. In addition, not only the key pathogen is important but also the bacterial species which naturally occupy this habitat. For establishing a database, a sufficient number of measurement repetitions should be performed. Here, the measurement strategy already defines the amount and variety of the bacterial strains under investigation. For bulk samples, the bacteria are cultivated prior to measurement. This allows for standardized conditions and can also lead to less complex datasets, since a pre-cultivation, e.g., on selective agar, might already preselect certain species of interest.

In contrast, if single-cell analysis is chosen, the database needs to be established with cultured bacteria. Here, as an alternative to real-world samples, the simulation of medical or environmental habitats might be necessary, in the form of spiked artificial samples, since Raman spectroscopy is very sensitive to changes in the growth conditions of bacteria. Afterwards, an isolation step may be required, and in the end the database is established following the same routine as the final environmental or medical sample will require. 

Finally, the last step of the study is the statistical evaluation. Here, calibration routines and pre-processing need to be established before a model database is trained. This model should, in the end, be challenged by independent validation data, and thereafter, it is desirable to use real-world samples for identification. 

Since all these aspects strongly interfere with each other, a reliable, reproducible, and stable database is strongly dependent on standardized techniques on all levels. 

All in all, many steps are required to generate a reliable Raman database for the identification of bacteria, and all are equally important for the quality of the study and the correct interpretation of the results.

## 2. Raman Spectroscopy

**Figure 2:**
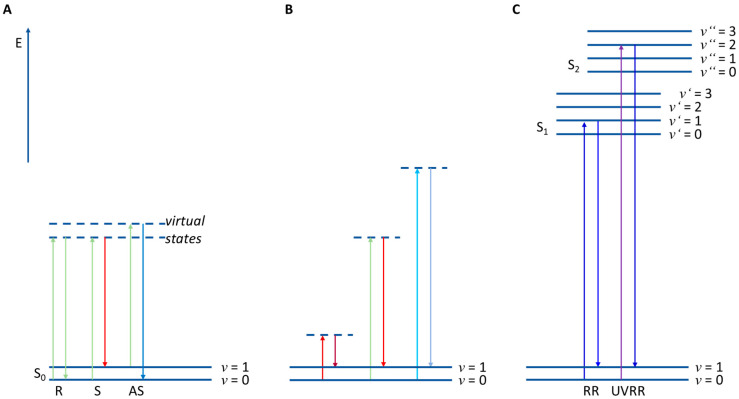
(**A**) Principle of Raman scattering: E—Energy; S_0_—electronic ground state; S_1,2_—first or second electronic excited state; *v*—vibrational eigenstates; R—Rayleigh scattering; S—Stokes–Raman scattering; AS—anti-Stokes–Raman scattering. (**B**) Stokes–Raman scattering with different excitation wavelengths. (**C**) RR—resonance Raman scattering; UVRR—UV resonance Raman scattering.

Raman spectroscopy is a scattering technique monitoring the vibrations of molecules. Here, the monochromatic light of the excitation laser interacts with a vibrating molecule, leading to spontaneous elastic and inelastic light scattering (see Figure 2A). The spontaneous elastic light scattering is known as Rayleigh scattering (R), whereas the inelastic light scattering as Raman scattering. This inelastically scattered light might be red- or blue-shifted and it is then referred to as Stokes- (S) or anti-Stokes–Raman scattering (AS), respectively. For normal Raman measurements the Stokes–Raman signal is used for spectral analysis since it is more intense than anti-Stokes–Raman scattering. In addition, the actual excitation wavelength normally does not change the energy (and, thus, spectral position) of the observed Raman signal (Figure 2B) [3,14,15].

Since Raman spectroscopy always samples all biomolecules inside the probing volume, most Raman methods are phenotypic which means the information of all biomolecules can be found in the Raman spectra of bacteria [15,16,17,18,19]. In principle, there are two types of Raman measurements of bacteria—bulk samples and single-cell measurements. For excitation in the NIR or UV regions, mostly bulk samples are used. In the NIR region the Raman spectrum requires a huge amount of biomass to receive a spectrum with a sufficient signal-to-noise ratio (S/N). In contrast, exciting with UV light leads to photothermal degradation of the sample; therefore, the laser is scanned over the sample. 

When combining Raman spectroscopy with a microscope, very good spatial resolutions < 1 µm can be achieved, allowing heterogeneous samples or even single bacteria cells to be analyzed with visible Raman excitation wavelengths [20]. By scanning over an area, or even in different layers, a larger sample can be examined, leading to 2D or 3D information of the sample. When bioorthogonal Raman labels with Raman signals in the silent region (1900–2600 cm^−1^) are used like, e.g., small molecules with C-D or -C≡C- groups, the contrast in 2D or 3D Raman images will further be enhanced [21]. In Raman microscopes, the excitation laser can also be used additionally as laser tweezers, which can be applied either in cuvettes or in special microfluidic devices, combining manipulation of the cell and analyzing it [22].

However, when the Raman excitation wavelength matches an electronic transition of a molecule, this resonance Raman spectrum (RR) achieves up to six orders of magnitude higher intensities than normal Raman spectra (Figure 2C) [10]. This can be used, e.g., to monitor special molecules in low concentrations in the bacteria like e.g., carotenoids [23,24,25] or cytochromes [26,27]. A special case is UV resonance Raman spectroscopy (UVRR). Here, excitation wavelengths below 260 nm excite molecules to a higher electronic state, leading mainly to resonance excitations of DNA/RNA bases or aromatic amino acids. This enables a genotypic-like Raman analysis, since with this technique the GC ratio can be detected [20].

A special method is surface-enhanced Raman spectroscopy (SERS). This method uses rough metal surfaces or metal nanoparticles to enhance the local field intensity in the immediate vicinity of the metal surface [28]. This approach therefore leads to signal enhancements of several orders of magnitude depending on the type of SERS substrate in combination with the excitation wavelength. Applying SERS to bacteria leads to two different approaches: either the bacteria are analyzed directly, or indirectly via an SERS tag with an SERS marker molecule [29,30,31,32,33]. In the first case, the SERS substrate will interact with the bacterial surface or secreted molecules and this information can then be used for identification or monitoring of physiological changes. In the second case, the SERS tag is bound to the bacteria surface via antibodies or aptamers and the specific label inside the used SERS tag then gives the spectral information to identify this bacterium [20,34,35,36]. For a more detailed description of SERS studies on bacteria please refer to references [29,30,31,32,33,37,38,39,40,41,42], since this manuscript is manly dedicated to conventional Raman spectroscopic studies. 

## 3. Strain Selection

**Figure 3:**
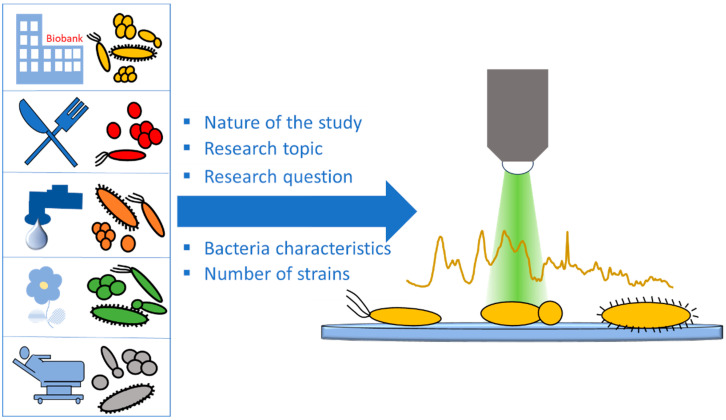
Schematic of species/strain selection.

Bacteria show a high diversity at an intraspecies level [43], with strains of the same species being adapted to different environments, and having different lifestyles, food sources, and metabolic characteristics [44]. Thus, in microbiological Raman studies, the selection of the appropriate strains is one of the most important parts of the study design. When this step is not given the necessary attention, the study outcome can be significantly influenced, leading to poor-quality results that are prone to misinterpretation and unable to provide the desired read-out. The appropriate strain selection is mainly related to the nature of the study, the study topic, and the specific research question asked (Figure 3). 

The goal of strain selection is to create a robust dataset that best represents the under-investigation topic and considers as many of its aspects as possible. These aspects depend on the research question and the study structure. When focusing on species classification, strains of the most frequent bacterial species in the under-investigation topic need to be included. In addition, adequate numbers of strains are also required for each group. However, the required number of strains is closely related to the nature of the study and the desired read-out. Proof-of-concept studies aiming to display the suitability of Raman spectroscopy as an analytical tool for a specific purpose can be performed with a small number of strains. When aiming at the evaluation of the classification accuracy provided by Raman spectroscopy and its comparison with gold-standard methods, larger datasets with multiple strains per group are required. Validation and test datasets on the other hand do not need to be large, since their purpose is to evaluate the robustness of the model [45]. Willemse-Erix et al. used four different strain collections of well-characterized *Staphylococcus aureus* isolates to perform epidemiological typing using Raman spectroscopy in comparison to PFGE. These results were validated using both historical isolates and prospectively collected strains [46], showing that Raman spectroscopy is a suitable tool for epidemiological typing. Nakar et al. used Raman spectroscopy directly on the culture dish to performed species classification on clinical isolates of the eight most common Enterobacteriaceae pathogens [45], covering a large spectrum of these groups’ representatives, including closely related species. In a study by Wang et al., 82 different strains of 18 *Acrobacter* species were used for species classification as well as differentiation from the closely related *Campylobacter* and *Helicobacter* genera [47]. In a similar study, Rebrošová et al. used 277 staphylococci strains from 16 different species for species classification [48]. These well-designed studies used large numbers of strains to indicate that the high sensitivity of Raman spectroscopy allows the differentiation of closely related species and genera that are sometimes difficult to differentiate even with conventional methods. When focusing on clinical isolates, it must be mentioned that in some cases the non-pathogenic bacteria from the normal flora also need to be included in order to cover the whole ecosystem, allowing for selective detection of the species of interest. 

When the study focuses on the presence/absence of a specific characteristic in a bacteria type, strains need to be chosen wisely to ensure that during data analysis the classification is based on the under-investigation characteristic and not on other, unrelated features. An example of such misleading results is the more intense signals of the carotenoid staphyloxanthin in methicillin-resistant *Staphylococcus aureus* (MRSA) strains when compared to methicillin-susceptible (MSSA) strains [49,50]. The higher average intensity of this molecule in the Raman spectra of MRSA strains does not necessarily apply to each strain of this group. Thus, it is an overstatement to consider the staphyloxanthin bands as a biomarker for the detection of methicillin resistance, especially since this molecule is a virulence factor and does not have any relation to the methicillin-resistance mechanism [51]. Another option for these types of strains could be label-free or label-based SERS, that allows the amplification of signals from specific bacterial molecules at low intercellular concentrations or without characteristic Raman signatures [37,52]. 

Another important factor that needs to be considered is the source the selected strains are obtained from. Different study types require different strain types, even when the species remains the same. When investigating cell mechanisms or proof-of-concept studies, bacterial strains purchased from commercial biobanks and strain collections can be used. Shen et al. used clinical isolates purchased from DSMZ, as well as patient isolates obtained from a clinical laboratory, to perform in vitro fiber-probe-based identification of pathogens in biofilms [53]. Since in both cases the isolates derived from patients, their combination in one dataset can be justified. Azemtsop et al. used a commercially available, environmental *E. coli* strain to investigate the incorporation of stable isotopes into bacterial cells [54]. In this Raman spectroscopic study, the only requirement on the strain was to be metabolically active. Thus, this fast-growing strain was enough to cover the experimental needs. In a proof-of-concept study of a new Raman setup, Maquelin et al. used ATCC strains and strains collected in a clinical microbiology laboratory to perform species identification of bacteria directly from their culture dish after 6 h of culturing [55]. The used ATCC strains were also strains originally isolated form patients and since they display similar growth characteristics as the clinical isolates the obtained dataset was homogeneous and allowed a proper study read-out. 

Other studies require strains from the same, topic-related origin, with shared characteristics, enabling their comparison. In a species classification study using *E. coli*, *K. pneumoniae*, and *K. oxytoca* isolates, Nakar et al. used 24 clinical strains deriving from the same hospital [45]. This provided the dataset with genetical homogeneity since the evolution of the different strains happened in the same environment. In addition, the restraint of the supragenome variability ensured the classification was performed on the species-related characteristics and not on other evolution-related factors that may dominate the differences in the Raman spectra. Similarly, Ghebremedhin et al. performed epidemiological typing with Raman spectroscopy compared to PFGE using 30 *Acinetobacter baumanii* isolates from the same department of an army hospital and from patients with similar wounds [56]. In a proof-of-concept study by Verma et al., commercially available *E. coli* strains were used as model organisms to investigate how treatment with sub-inhibitory concentrations of bactericidal and bacteriostatic antimicrobial agents affects their Raman spectral signature [57]. The results were validated with two of the strain’s mutants that displayed a relatively high and a relatively low resistance towards these antimicrobials, ensuring an isogenic background and avoiding distortion of the results.

When using Raman spectroscopy for the development of laboratory methods for specific tasks, the study design could require the bacteria to be within the specimen they are inhabiting in nature. In the study of Rusciano et al., Raman spectroscopy was applied to sputum samples from patients with cystic fibrosis for the detection of *Pseudomonas aeruginosa* and *S. aureus* infection [58]. This allowed simple and painless sampling for the detection of the two most common pneumonia-causing pathogens in this patient group. In other cases, and due to access difficulties as well as restrictions on real-life samples and their use in proof-of-concept studies, a simulation of the natural habitat may be required in vitro. To develop a cultivation-free and Raman-compatible isolation method of bacteria from bloodstream infections, Lorenz et al. removed hemoglobin from sheep blood previously spiked with strains purchased from DSMZ [59]. In this first step, it was shown that the isolation method was Raman compatible, setting the foundation for further testing on blood cultures from patients. Similar, Dekter et al. mimicked the culture conditions of a positive blood culture by incubation of *Enterobacter cloacae* patient isolates in a used blood culture flask. The purpose of this was to develop an antimicrobial susceptibility test (AST) for ciprofloxacin using Raman spectroscopy [60]. In another proof-of-concept study by Kloß et al., Raman single-cell analysis was applied directly on spiked urine. Following the same principle, the urinary tract infection (UTI) was simulated by inoculating bacteria pathogens, obtained from DSMZ and ATCC, into sterile filtered urine [61]. Shen et al. used a drip flow reactor to create a biofilm on CaF_2_ slides, followed by Raman spectroscopic analysis of the bacteria, as well as the extracellular polymeric substances (EPSs) the biofilm was composed of [53]. This simulation provided insight on the fundamental structure of the biofilm and can be further used on the analysis of natural biofilms from different origins.

When specific characteristics of the bacteria are to be investigated, it could be that the required bacterial strains are not available either in nature or in biobanks. In this case, the strains need to be artificially created in the laboratory to meet the criteria of the study. Examples of this are the studies of Germond et al., Saikia et al., and Walter et al., where transformation was used to create isogenic strains with and without specific antimicrobial resistances [62,63,64]. In these studies, the high sensitivity of Raman spectroscopy allowed the differentiation of isogenic strains carrying resistance genes to specific antimicrobial compounds. Another example of laboratory-induced characteristics in strains for study design purposes is the study of Yang et al. [65]. Here, antimicrobial resistance to ampicillin was gradually introduced in situ into the *E. coli* K12 standard strain after 10 cycles of antibiotic exposure followed by cultivation in an antibiotic-free environment. This allowed the investigation of the evolution of antimicrobial resistance in the evolved populations after each treatment cycle, using D_2_O labeling as a marker for metabolic activity and single-cell Raman analysis. 

Another important factor for strain selection is the number of strains per group that are included in the study. The created dataset should be large enough to allow a cross-validated statistical evaluation. The number of strains and spectra measured depends on the type of the cross-validation (CV) that is to be performed. For leave-one-batch-out CV, several biological replicates of the same bacteria need to be obtained at different time points. For leave-one-strain-out CV, as many strains as possible should be included in each group. For 10-fold CV the number of spectra obtained needs to be high, to include as many spectra as possible in each created sub-division. This method, however, is not recommended for classification studies since it can easily result in overfitting of the statistical model. Further insight on statistical evaluation, however, will be provided in the following sections of this review. 

When strain selection is performed according to these criteria it is ensured that the study design is in line with the asked research question. This leads to a high-quality study that can provide the desired results read-out while minimizing the possibility of misleading and inconclusive outcomes and maximizing the results’ accuracy. 

## 4. Principal Factors Influencing Raman Measurements

**Figure 4:**
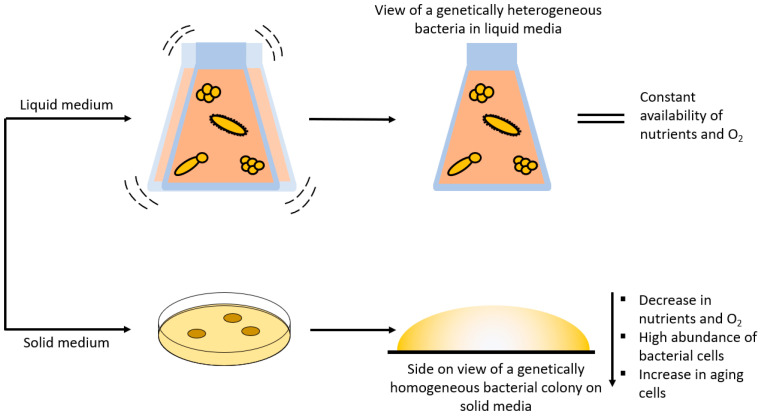
Schematic workflow for liquid and solid media and their influence on cultivation conditions.

Raman spectroscopy combined with chemometric evaluation is utilized as a phenotypic method to characterize biological samples. This method is especially relevant when it comes to identifying and monitoring pathogenic and non-pathogenic bacteria [8,66,67]. Here, the phenotyping is based on the entirety of the biomolecules of the bacteria that are captured in a Raman spectrum. What makes this method so interesting is that it is cost-effective, rapid, label-free, and is mostly not obscured by water molecules. However, biological samples (i.e., bacteria) are prone to change their phenotype based on environmental or genetic changes [5]. Environmental changes could be the short- or long-term transportation from one point to another, or even plain storage in a fridge or freezer [68,69]. Factors such as the cultivation medium [70,71,72], the cultivation temperature [71], cultivation time [6], or even the CO_2_ and O_2_ levels [73,74,75,76,77] may have an impact on the phenotype of the bacterium. A genetically homogeneous colony on a Petri dish may result in a totally different outcome than a genetically heterogeneous liquid culture (Figure 4). Lastly, it is also important to consider with which excitation wavelength the bacterial samples are excited to achieve the desired outcome, and whether single-cell, or bulk analysis, is performed. Especially for single-cell analysis the cell-to-cell variance should be considered [18]. In summary, these are just some of the many factors that may influence the bacterial phenotype and subsequently the Raman spectrum and chemometric evaluation [8].

### 4.1. Storage and Transport Conditions

The success of Raman spectroscopic identification of bacteria depends on the quality of the created database and its chemometrics. As mentioned above, when a new database is to be established, several considerations must be made, and possible obstacles must be overcome. Naturally, the Raman spectra of the investigated strains/species/genera must be included in the to-be-established database. Unfortunately, before any samples can be measured, they need to be shipped or transported from the sampling site or strain collection to the laboratory. Even then it is not guaranteed that the samples will be measured immediately, hence non-biological factors such as time and storage can influence the Raman spectra significantly [68,69]. In a comprehensive study conducted by Wichmann et al., they investigated the influence of low storage temperatures on the quality of Raman spectra. Here, five aliquots of a bacterial liquid culture were taken, and the first one was directly measured (control) while the others were centrifuged, pelleted, and resuspended in 0.9% NaCl to simulate a clinical sample. The second aliquot was measured immediately after resuspension, whereas aliquots three to five were kept at 4 °C. After 24 h, aliquot three was measured and aliquots four and five were stored at −80 °C. Samples four and five were thawed at room temperature and measured after 7 and 30 days, respectively. By collecting the Raman spectra and evaluating them with chemometrics, subtle differences could be detected throughout all the bacteria and storage conditions. Therefore, they concluded that the longer bacteria are stored, the worse the identification becomes [68].

### 4.2. Cultivation Conditions

For Raman spectroscopic experiments, it is very important to decide whether the bacteria should be cultivated on solid media within Petri dishes [70,71,78,79] or in liquid media [72,79,80,81] within a flask. Both cultivation techniques have their respective advantages and disadvantages (Figure 4). It is more likely to find single bacterial colonies on solid media than in liquid media. These single colonies derive from one single replicating bacterium, which means that the colony itself is genetically homogeneous. In addition, a gradient containing several parameters (age, nutrients, CO_2_ and O_2_ levels, etc.) can be seen within single colonies [71]. These parameters can then be further influenced by the type of cultivation media, temperature, humidity, and so on within the incubator. Harz et al. cultured several *Staphylococcus* species under the suggested conditions (i.e., media and temperature) and under different conditions (e.g., 30 instead of 37 °C) to examine if the cultivation circumstances influenced the identification procedure in any way. Surprisingly, the different growth circumstances modified the bacteria; yet, using Raman spectroscopy with a support vector machine is an exceptionally competent approach for identifying single bacteria at different cultivation conditions, not only at the species level but also at the strain level [71].

In liquid media, the bacteria grow in a suspension and not in colonies, which means that the bacteria are genetically heterogeneous, yet the same parameters apply (Figure 4) [72,82,83]. Additionally, liquid cultures are further influenced by (non-)shaking and the speed of the shaker in the incubator, since shaking influences the amount of dissolved oxygen in the medium [83]. In this study, *Clostridium acetobutylicum* was grown anaerobically in liquid media without shaking, and Raman microscopy was used to gather spectral data on the chemical composition of the cells and their heterogeneity in liquid media as well as to identify subpopulations with various cell compositions and physiological traits [72]. Yamamoto et al. presented a growth/no growth prediction method for 21 bacterial strains grown in liquid media under different combinations of sodium acetate and low temperatures that implements the use of Raman spectroscopy and machine learning. The conditions of the cultivation process were two incubation temperatures (5 and 10 °C), three sodium acetate concentrations (0, 0.25, and 0.50% *w*/*v*), and eight incubation time periods (0, 1, 2, 3, 4, 5, 6, and 7 d). The cells were then suspended in TSB broth (10^4^ cfu/mL) with 100 µL aliquots in each 96-well plate at a starting OD_600_ of 0.1 and measured each day (>0.1 = growth; <0.1 = no growth). The machine learning model was able to predict the growth or no growth response of 21 unknown bacterial strains with 90% accuracy in liquid media by extracting the spectral information from previously known bacteria [84]. In conclusion, when it comes to solid and liquid media, changing the culturing environment alters the biochemical makeup of a microbial cell, which can affect the discrimination process, which should aid in identifying the studied species [70,85,86].

### 4.3. Time

Time is and will always be an important factor in bacterial cultivation since the cells age and die over a certain period. This is supported by the fact that bacteria go through several phases in their life cycle: the lag phase, the log or exponential phase, the stationary phase, and the death phase [83]. To identify which phase the bacteria are now in and when it is optimal to use Raman spectroscopy to assess them, a growth curve must be developed [83]. It has been previously shown that the age or the growth phase of the culture not only have an impact on biological variation but also on the Raman spectra [34,69,78,79,87,88]. In a study conducted by Stöckel et al., growth curves (0–130 h) of pigmented *Mycobacterium aurum* and non-pigmented *Mycobacterium smegmatis* were determined to analyze their growth behavior. After knowing the corresponding times at which the growth phase occurs, Raman measurements were performed in the exponential and in the stationary phases. Both species revealed normal bacterial Raman bands during the exponential phase, but during the stationary phase *M. smegmatis* displayed lipid-associated Raman signals (mycolic acids) while *M. aurum* displayed signals from carotenoid-like compounds. Because pigments are extremely sensitive to conformational and structural changes, this behavior must be considered when using Raman spectroscopy to detect and identify pigmented bacteria [23,24,87,89].

### 4.4. Atmospheric Gases

Since Raman spectroscopy is a phenotypic method, another culture condition that affects the growth of bacteria and subsequently the Raman spectra is the CO_2_ and O_2_ levels in the incubator. The level of CO_2_ in medical samples varies not only across various body parts, but also, for example, in the case of the lung, with the course of disease [90]. Due to changing CO_2_ levels, bacteria have adapted to varying CO_2_ concentrations by different phenotypic variance [83]. *Escherichia coli*, for example, has the ability to change its metabolism from glycolysis to fumarate respiration depending on if CO_2_ is added or not. This ability was exploited in a study by Wichmann et al. by slowly increasing the CO_2_ concentration (0%, 4%, 6%, 8%) in the incubator to determine the exponential phase. By increasing the CO_2_ concentration, it was possible to observe the switch in the metabolic pathway by Raman spectroscopy [74]. Therefore, it is quite possible that Raman spectra of the same bacteria may phenotypically differ with fluctuating concentrations of CO_2_. On the other hand, oxygen deprivation can also influence the Raman spectra. In a proof-of-concept study by Kniggendorf et al., confocal Raman microscopy was applied to ammonia-oxidizing bacteria (*Nitrosomonas* and *Nitrosospira* species) that were subjected to aerated and oxygen-deprived media caused by either shaking or not shaking the flask. This resulted in the same Raman bands as cytochrome c, yet cytochrome c was found to be elevated in the oxygen-deprived cells. Furthermore, cells with a high ferrous cytochrome c concentration were observed in deprived *Nitrosomonas eutropha* and *Nitrosospira* samples, which might indicate continuous electron storage at the time of assessment [73].

### 4.5. Media Composition

**Figure 5:**
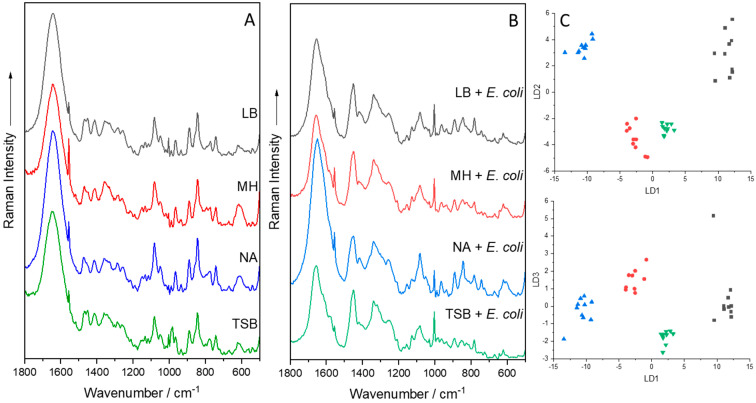
Mean Raman spectra of various agar, either pure (**A**) or with *E. coli* (**B**), measured with a Raman fiber probe at 785 nm. PCA-LDA plot of *E. coli* grown on various agar (**C**). Each dot represents one Raman spectrum. Black: Luria/Miller agar (LB); Red: Müller–Hinton agar (MH); Blue: Nutrient agar (NA); Green: Tryptic Soy Broth agar (TSB).

Differences between laboratory and real-world conditions must be addressed when constructing a Raman spectral library. Cultivation of microorganisms is carried out under controlled settings to stimulate and promote perfect bacterial growth. In contrast, the human body can be seen as a growth medium in which the conditions may differ and not promote optimal bacterial growth. To explore in which capacity growth media and growth phases influence Raman spectra, *Mycobacteria* were cultured on specific (Middlebrook and Kirchner) and on non-specific (brain heart infusion and lysogeny broth) media. As the mycobacteria aged, the mycolic acid bands decreased in intensity, while the carotenoid bands increased. As for the different growth media, the classification accuracy was 97.2%, which indicates a discernible spectral variance between the mycobacteria grown on different media. These variances are crucial in this respect since they may influence the predictive model [78]. Furthermore, if growth medium must be produced from many separate components, such as salts, these components may also alter Raman spectra (Figure 5). To test this, *Synechocystis* cells were grown in the presence of acetate (7.5–30 mM), NaCl (50–150 mM), and MgSO_4_ (0–62.5 mM) in BG11 media. After Raman measurement and chemometric analysis, very distinguishable clusters were observed based on the phenotypic response induced by the added external stimuli. These phenotypic changes were a result of a change in amino acids and fatty acids induced by the limited salt content [91].

### 4.6. Matrix Simulation

Since the parameters of a certain habitat have a large influence on the respective bacteria phenotype, these parameters need to be replicated as closely as possible within a laboratory setting. Therefore, “real-world samples”, like patient or environmental samples, present one of the biggest challenges since the growth of the bacteria depends on the matrix composition, temperature, time, pH value, and others, which will in turn affect the Raman spectra. The culture parameters should ideally be as near to the actual sample as achievable, although this is only attainable to a certain degree under laboratory conditions. Some of these challenges can be overcome by producing and measuring many batches of each strain or species. Additionally, the origin of the sample (sample matrix) needs to be simulated so that the experimental parameter is as close to the original as possible. Simulating the “real-world” sample as closely as possible will provide more information on the internal and external parameters that may influence the Raman spectra. 

To simulate the origin of a certain sample, be it environmental or medical, the bacterium of interest must first be isolated or bought from a strain collection/research hospital. Next, it is important to replicate the matrix composition in which the bacterium is embedded as accurately as possible. For example, to examine bacteria in real sputum samples, the sputum needs to be simulated by using a recipe for artificial sputum [50,58,92,93]. Here, the artificial sputum was mixed with a bacterial suspension to artificially replicate a real sputum sample. The bacteria were then subjected to several isolation steps to determine the isolation yield, Raman measurements were performed, and the Raman spectra were evaluated by means of machine learning [58,94]. For comparison, to simulate ascitic fluid, bacteria were first grown on solid media and then transferred to sterile filtered ascitic fluid for further incubation. The ascitic fluid culture was subsequently put through a number of separation processes, Raman measurements, and a chemometric evaluation of the Raman spectra [94,95]. 

To add another layer of complexity, Meisel et al. examined spiked meat (minced beef and chicken breast) for meat-associated pathogens via Raman spectroscopy. Bacteria were selected based on their commonality as food-borne pathogens and were cultivated on meat-like media (Columbia blood agar, brain heart infusion agar, and Müller–Hinton agar). Commercially available fresh vacuum-packaged minced beef and chicken breast were then spiked with 1 mL of bacterial suspension. The spiked meat was incubated at 4 °C for 24 h to let the bacteria adapt to and comfortably flourish in the new matrix. To isolate the bacteria from the meat, the meat was shaken in a rotator and the resulting meat juices were filtered, centrifuged, and washed. The resulting bacteria pellet was then measured, and the Raman spectra were evaluated [96]. Of course, these are just a few examples of how vibrational spectroscopy can be utilized to rapidly identify microorganisms from complex matrices [97,98,99,100,101,102,103,104,105].

Additionally, the composition of matrices in which bacteria are embedded can also be very simple such as water, urine, or blood. Water [106,107,108] and urine [61,109,110,111,112,113,114] samples are both clear liquid substances that can be spiked with bacteria of interest and which can then be isolated by centrifugation, filtration, and washing steps. It is also possible to spike artificial urine and apply single-cell Raman spectroscopy to identify the pathogenic bacteria [115]. Another approach presented a fast and reliable method for identifying bacterial pathogens in primary urine samples from patients with suspected UTIs [109]. Blood, on the other hand, is a whole different matter. Here, blood is nigh on impossible to simulate, hence animal blood such as sheep or horse blood is required. The blood can then be spiked with a bacterial suspension. The isolation process itself involves several steps including lysis via Triton X-100 solution and enzymatic digestion of the hemoglobin via Pronase E [59,116,117,118,119]. Nicolaou et al. employed two approaches (FTIR and Raman spectroscopy) to identify and count *Staphylococcus aureus* in spiked ultra-heated milk, as well as to investigate the growth interaction of *S. aureus* and *Lactococcus lactis* [120,121].

### 4.7. Influence of the Raman Setup

In principle, there are two main Raman techniques to consider: single-cell analysis [6,51,54,59,68,74,105,115,122,123,124,125,126,127,128] and bulk sample analysis [45,51,129,130,131,132,133,134]. Each comes with their own advantages and disadvantages. If you have a limited amount of time and want to obtain results quickly, single-cell analysis is the way to go. In summary, this technique includes an isolation stage that does not kill or change the causal pathogen from its original matrix, followed by an identification step. However, the isolation procedure must be adapted to the matrix from which the sample is taken since the matrix can be a simple (e.g., water [106]) or a more complicated one (e.g., bronchoalveolar lavage fluid (BAL) [105], sputum [135], or blood [59]) depending on the location of the desired sample. In stark contrast to the single-cell analysis method, bulk samples can only be measured and identified from pure samples that have been previously cultivated. Typically, a thick bacterium suspension or high amounts of biomass on solid media is deposited onto a substrate (e.g., CaF_2_) and then dried to form a homogeneous layer. Although this approach is more time-consuming than single-cell analysis, it is significantly more accurate in terms of discrimination and identification [51,132]. Also, due to the large amount of biomass required, the cultivation step can easily be standardized [129]. 

The huge disadvantage is that due to the high amount of biomass, large quantities of fluorescence can occur, which will most likely obscure the Raman spectra. To avoid this outcome, excitations in the NIR or UV regions are used. NIR excitation produces lower-intensity Raman bands, yet the sample stays undamaged and sum spectra can be collected from heterogeneous samples. Alternatively, excitation in the UV region (<260 nm) results in a fluorescence-free spectrum while also enhancing Raman signals of DNA/RNA bases and aromatic amino acids, leading to a higher signal-to-noise ratio. Most importantly, if a sample is measured with a UV setup, it is prone to damage the sample since complex molecular structures break down and burn [51]. To mitigate this photodegradation, the sample can be rotated manually in the *x*,*y*-directions [136], or an automated system needs to be employed that rotates the sample automatically, where the gear system moves the sample table with an offset to prevent measuring the same spot twice [54,124,137]. By applying this setup, it was possible to characterize isotopically labeled *Escherichia coli* cells and monitor their overall metabolism as well as metabolically active cells [54]. It is also quite useful to monitor cellular metabolic activity in *E. coli* with ^18^O incorporated into the amide I group of proteins and DNA/RNA bases [124]. As previously mentioned, when choosing a wavelength in the deep UV region, the chances are high that nucleic acid and aromatic amino acid signals are enhanced. Obviously, there are many more studies that have been conducted using UV resonance Raman spectroscopy and analyzing bacterial samples [51,112,130,132,138,139,140,141,142,143].

In contrast, when measuring at a 785 nm excitation wavelength, a different Raman setup is needed. For instance, a high-performance Raman module was coupled to a custom-built inverted microscope stage with an automated *x*,*y*-stage and operated with specialized software. The microscope itself had a custom-designed microscope objective suited for Raman investigations in the wavelength range of 750–1000 nm, which is optimized for air-dried bulk samples on fused silica glass slides. By utilizing this setup, it was not only possible to measure bulk samples of different *Mycobacteria* species, but also to investigate if heat-inactivation changed the spectral features compared to Raman spectra of viable *mycobacteria* [144]. At the same time, it is possible to apply this method for real-time typing of 118 *Staphylococcus aureus* isolates as well as to classify stored (−80 °C) and methicillin-resistant *S. aureus*-colonized individuals [46]. Furthermore, isolates of methicillin-resistant coagulase-negative staphylococci were used to evaluate Raman spectroscopy as a typing tool and to investigate the diversity between colonies with identical and different morphologies [145]. Naturally, many studies have been employed that use an excitation wavelength of 785 nm when characterizing bacteria [49,104,116,133,146,147,148,149,150,151,152].

A different approach used to acquire Raman spectra at an excitation of 785 nm was to use a Raman fiber probe coupled with a high-performance Raman module. The Raman fiber probe was then applied to collect Raman spectra from immersed biofilms on CaF_2_ slides in saline solution. This setup not only made it possible to measure Raman spectra of immersed biofilms from embedded bacteria and yeast cells but also from the surrounding extracellular polymeric substance matrix [53]. The Raman fiber probe was also applied to directly measure colonies of nine clinically relevant microorganisms. Here, it was important that the microorganisms were measured directly from stainless steel Petri dishes since these do not give unwanted background signals compared to the commercially available standard Petri dishes [153]. Speaking of which, it is never a terrible idea to measure the background such as the Petri dish or the medium since this spectral information might explain or alleviate unwanted signals, artifacts, or headaches (Figure 5) [6,8,104,154]. Figure 5 also shows the effect of different media either in their pure solid form or cultivated with *E. coli*. To highlight small variations in the spectra, a PCA-LDA was implemented, in which the optimal outcome was a small variance within each individual group but a large variance between the four investigated groups. The model revealed exactly this outcome.

In summary, it can be said that not only external, but also internal biotic and abiotic factors may influence the bacterial phenotype and ultimately the outcome of the Raman spectra as well as the chemometric evaluation. In addition, when it comes to simulating a real-world sample (environmental or medical), then it is advisable to consider all the parameters which make up the sample including the composition of the matrix, the typical indigenous bacteria, and the cultivation parameters. Furthermore, it is important to consider with which Raman setup and at which excitation wavelength the desired results can be achieved [6,8,104,154].

## 5. Isolation of Bacteria

**Figure 6:**
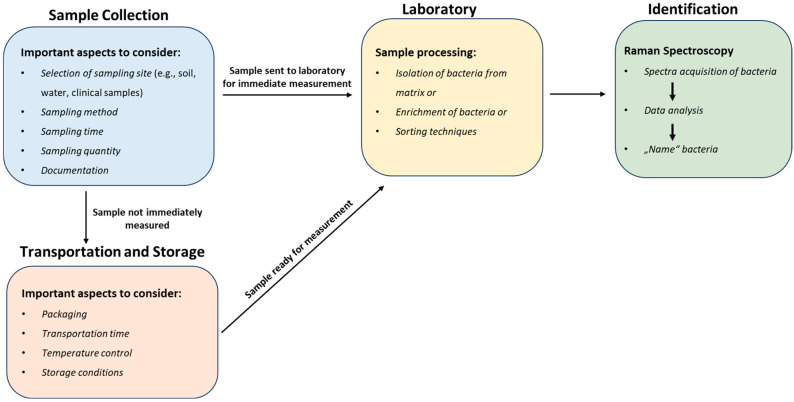
Integrated workflow for bacterial isolation: sample collection, transport and storage, processing, and Raman spectroscopy identification.

The ability to isolate and identify bacteria provides us with a window into their complex world, allowing us to understand their ecological dynamics, genetic makeup, and impact on our lives, which has contributed to significant advances in food safety [7,96,152,155,156], medical diagnosis [8,14,20,59,130,157,158], the implementation of effective environmental preservation measures [159,160,161,162], and the revolution of industries through biotechnological advancements [163,164,165,166,167]. Contrary to the common perception of bacterial isolation as a simple task, the effectiveness of the isolation strategy for Raman spectroscopy analysis is dependent on a complex array of factors, including proper specimen collection, transport, storage, and processing. This is because Raman spectroscopy is a phenotypic characterization tool that analyses the entire cell; as a result, any influence on the cell could alter the spectra, potentially leading to hampered bacterial identification. In this section, we highlight key areas and propose some solutions to improve the reproducibility and reliability of a bacterial isolation strategy for subsequent analysis using Raman spectroscopy (Figure 6).

### 5.1. Sample Collection

The first step in the bacterial isolation process is the collection of the sample. The effectiveness and integrity of the ensuing isolation and identification processes are largely dependent on this stage [168,169,170]. The technique of sample collection may vary depending on the source of the sample, whether environmental (soil, air, or water) or anatomic sites such as wounds or mucosal surfaces, bodily fluids, bone and tissues, the specific bacteria being targeted, and how the sample is going to be processed. It has been well established that Raman spectra and bacterial classification vary depending on the growth media, growth phase, intrinsic storage material, and cultivation conditions of bacteria [70,71,78,87,171,172,173,174]. Similarly, clinical and environmental samples contain bacteria at various stages of development and exposed to varying nutrient loads, which also affects Raman spectra [68,78]. These changes may have an influence on the discriminant models used to classify bacteria, potentially leading to misclassification [68,70,78,87,171]. Thus, understanding the impact of growth phase and medium on classification models, as well as how Raman spectra change because of these variables, is key to proper sampling. Sample collection must be approached meticulously to ensure the preservation of the bacterial diversity present in the original environment [170]. Several axiomatic principles govern the collection of samples for microbiological testing [168,169,170,175,176,177,178,179,180]. 

The first of these rules is the most obvious: the specimen must be collected through strict aseptic procedures to minimize sample contamination [168,169,170,175,177,178,179,180]. Raman spectroscopy is a phenotypic characterization; thus, any foreign substances or contaminants introduced into the specimen can distort or mask the true Raman signal from the bacteria of interest. Contaminants can affect the Raman spectra in various ways, such as weakening Raman signals, fluorescence, introducing additional background noise, or creating interference.

Secondly, depending on the precise place being sampled it is crucial to select the best sampling strategy. For instance, swabbing or aspiration techniques may be employed for anatomic sites, such as wounds or mucosal surfaces [168,170,175]. Environmental sites (water, soil, or air), on the other hand, may require surface sampling or water filtration methods utilizing sterile containers or specialized collecting apparatus designed for environmental sampling [180]. Additionally, it is important to collect an adequate and representative sample by sampling from multiple sites if necessary and taking into account the spatial distribution of bacteria, sampling time, and also the average environmental conditions to ensure a comprehensive assessment [180,181,182]. For “artificially” inoculated samples, bacterial cell harvesting should be performed at defined time points since the age or growth phase of the culture also impacts biological variations in the bacteria, and hence, in the spectra [87,105]. Ideally the appropriate time should be during the exponential phase when the bacterial culture has reached its maximum density but has not yet entered the stationary phase. This timing ensures the highest biomass and metabolically active cells [78,171]. 

Thirdly, if possible, sufficient material must be submitted for cultures and other tests. When it comes to sample volume, it is always best to extract the largest possible amount from the most infected regions or area of interest [168,169,170]. 

Lastly, proper labeling and documentation of samples, including relevant patient or environmental information, is also critical for accurate identification and interpretation of the results [168,169,175]. When filling out specimen request forms or using automated order entry systems, collectors should be as detailed as possible. Key details include the site(s) of specimen collection, the patient’s antimicrobial therapy status, the specific pathogens sought, the specimen collection methods, and potential hazards to laboratory personnel [170].

### 5.2. Transportation of Samples and Storage Conditions

Transport and storage are two of the least controlled aspects in microbiological testing, yet they are frequently overlooked; therefore, their impact on the Raman spectra of bacteria have not been sufficiently investigated [68].

*Transport:* Typically, specimens for microbiological testing are transferred in sterile containers or, in the case of fluid specimens from medical samples, in the syringe used to collect them [169,170,175]. Others are transported in transport media, which, in spite of their preservative properties, do not reflect the in vivo conditions of the “clinical” patient specimen and could lead to extreme loss of vulnerable bacteria [176]. Although it is a widely known fact, clinical patient specimens exhibit considerably higher losses of bacteria than artificially inoculated samples [183,184]. The same is true for environmental samples, as once a sample has been collected from the field its microbial populations are prone to changes regardless of the storage method used [180]. Nevertheless, specimen containers must be carried in such a way that no damage is caused to the sample and staff exposure to blood or other bodily fluids is avoided [169,170]. Generally, it is expected that the specimen for culture or bacterial analyses should be transported to the laboratory as promptly as possible for processing [168,169,170,175,176,177,180]. This is particularly important for vulnerable pathogenic bacteria, such as clinical anaerobes, *Shigella* spp., *Neisseria meningitidis*, *N. gonorrhoeae*, *N. pneumococci*, *Haemophilus influencae,* and β-hemolytic *Streptococci*, which die off quickly, usually within 2–4 h of storage [168,169,176,177,184]. In the case of long storage times of much more than 4–5 h, robust bacteria are not only overlooked, but it becomes impossible to determine with any certainty whether the isolated bacteria is an indigenous flora or the potentially pathogenic agent being targeted [177,184]. Most specimens can be transported at room temperature, while some require transportation on ice or in a cooling box within a temperature range of 2 °C to 8 °C [168,169,170,175,176,180]. Lastly, specimens submitted for culture of *Neisseria* species should be transported in an atmosphere with sufficient CO_2_ and humidity and in a manner that prevents wide temperature fluctuations [168,170].

*Storage:* Most specimens require prolonged storage prior to processing, while in certain cases samples may be kept for 48 h or longer due to logistical constraints, necessitating refrigeration or freezing, generally at 4 °C, or at temperatures ranging from −20 °C to −80 °C as required [168,169,170,176,180,185]. Refrigeration preserves the bacteria’s viability and relative proportions, which are critical for semiquantitative or quantitative culture (e.g., cultures of sputum or urine) interpretation [168,170,176]. Refrigeration additionally minimizes the proliferation of contaminants. Specimens that should not be refrigerated include blood, bone marrow, nasopharyngeal aspirate, and cerebrospinal fluid (CSF), which should be kept at room temperature or in an incubator at 35 °C to 37 °C [168,169,170,175]. However, the longer bacteria are stored, the worse the identification becomes according to Wichmann et al. [68], who investigated the influence of transport and storage at low temperatures on the bacterial Raman spectra of *S. cohnii*, *S. warneri*, *L. innocua*, *E. coli*, *K. terrigena*, and *P. stutzeri*. With just a look at the mean spectra, only *S. cohnii* exhibited obvious differences under the different storage conditions, but chemometric methods revealed changes across all bacteria. Therefore, when planning long-term experiments, it is particularly important to consider the impact of transport and storage on the Raman spectra of bacteria. Ideally, a training dataset should be created under the same exact conditions that bacterial samples would be subjected to later to minimize misclassification or classification errors [68].

### 5.3. Sample Processing: Isolation from Matrix and Isolation Strategies

Raman spectroscopy, as a phenotypic method, can detect cellular adaptations of bacteria in response to environmental fluctuations [14,105]. Consequently, the resultant Raman spectra are sensitive to various parameters, and this is particularly pronounced within environmental and patient samples where bacteria are typically embedded in a matrix of some sort [105]. “Matrix” in this context refers to all other particles or molecules, organic or inorganic, besides the target particle or molecule, in this case bacteria cells [59]. However, the prerequisite for Raman microspectroscopy is a destruction-free isolation of bacteria cells without matrix interference, which could otherwise contribute to the spectra [135,186] or hinder the microscopic location or observation of the cells [59]. Of course, other aspects such as (I) cell concentration in the sample [6,187]; (II) yield of cells [6,105]; and (III) minimizing any influence on the diversity within a bacterial species [186] are also important. To date, numerous studies have isolated bacteria from a wide range of matrices, from medically relevant media such as bronchoalveolar lavage (BAL) [105], ascitic fluid [94], blood [14], urine [188], and sputum [135,157] to consumables such as milk [155,186], meat [96], and feedstuff [125]. These featured various Raman-compatible isolation strategies of varying complexities that may be considered culture-dependent or culture-independent. The methods include density gradient centrifugation, culture-independent filtration, optical trapping, dielectrophoresis, immunocapture, and matrix digestion [59,105,113,132,157,186,187,188,189]. However, in most of these examples, due to the complex nature of the samples, a combination of other enrichment processes proved to be effective. For example, filtration combined with centrifugation [188] or the combination of filtration, centrifugation, and dielectrophoresis [190] is highly effective for diagnosing urinary tract infections and can detect even low levels of cell concentration. Nonetheless, some of the published strategies are still in the proof-of-concept stage and have not been fully optimized. Alternatively, Raman measurements can be conducted directly on bacteria present in the matrix [6]. This section covers such options and presents an overview of Raman-compatible isolation methods for detecting bacteria in complex matrices. It also offers useful tips on effectively carrying out these methods and common mistakes to avoid. Furthermore, these methods have limitations that are usually overlooked, so we discuss both the suitability and limitations of the techniques here. 

*Cultivation:* The classical approach for obtaining bacteria cells from samples is, of course, culturing [191]. This technique allows for the Raman spectroscopic analysis of large amounts of biomass, enabling measurements to be conducted in the bulk phase [6,132,153,192,193]. Usually, a small amount of sample is inoculated on a solid or liquid growth medium that provides nutrients necessary for bacterial growth, and then, incubated under regulated conditions, e.g., temperature, humidity, pH, oxygen availability, CO_2_ concentration, illumination, and agitation [191]. The bacteria are then harvested from the medium, and then, centrifuged to generate a cell pellet, which is subsequently deposited on a Raman substrate [194,195] for Raman measurements when the material has dried [61,135,158]. For the isolation of pure cultures from ‘real-world samples’, several cultivation conditions might affect the Raman spectra and hence the bacterial species characterization. Therefore, ideally, the cultivation conditions of the bacterial cells for the database should be as close as possible to the environment in the actual samples and must be kept consistent [6]. It is also essential to note that the same medium in liquid or solid form can result in different phenotypes of the same bacterial species depending on the nourishment they receive [196]. As a result, spectroscopic differences can arise, with some bacteria being distinguished spectroscopically much better after cultivation in liquid media than after cultivation in a solid medium, which must all be considered [81]. Certain media or media supplements generate their own Raman signal, thus must be avoided [59,196,197]. Finally, the appropriate time for taking samples for analysis is crucial, as the age or growth phase affects biological variations [87,105]. In the end, not all spectroscopic laboratories are biosafety zones. In these cases, appropriate inactivation strategies are required which ensure safe sample handling and minimal sample alteration [108,155,158,198,199,200].

However, this technique has a limitation in that the culturing stage might take many hours or days, which can be crucial for some applications. Additionally, conventional methods cannot be used to culture all bacteria since only a small proportion of the overall diversity in nature can be grown in the laboratory [191,201,202,203].

*Filtration:* Filtration is a physical method that can be used to separate bacteria cells from other particles or fluids by passing them through a porous membrane/barrier [191,204]. The membrane acts as a sieve, retaining bacteria cells on its surface; thus, depending on the pore size of the filter, cells up to a certain size can be removed specifically. Bacteria, which typically range in size from 0.5 to 5 μm, can be separated from eukaryotic cells via filtration because they are significantly smaller, thus providing the necessary enrichment of a sample [6]. 

**Figure 7:**
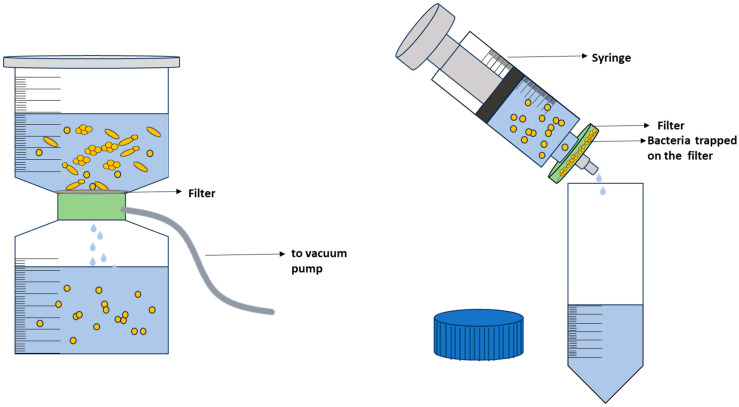
Different filtration techniques.

In simple filtration methods, membrane filters are commonly used, where a syringe or pump is used to force the fluid through a filter and into a sterile collection vessel (Figure 7) [83,180,205]. There are various types of membrane filters, with porosities typically ranging from 0.22 µm to 0.45 µm, such as polycarbonate, polytetrafluoroethylene, polyvinylidene fluoride (PVDF), and nitrocellulose [83,206], each with unique properties and suitability for bacterial filtration. Nevertheless, the most important consideration is that the pore size of the filter membrane should be small enough to capture the bacteria of interest, but not too small to clog the filter or damage the bacterial cells. Consequently, the filter membrane should be selected based on the expected size range of the target bacteria [180]. In some cases, a sample may contain an excessive number of cells or particulates, which can hinder the filtration process. To address this issue, pre-filtration steps may be necessary, such as using filters with larger pore sizes to remove non-bacterial particles [125,135,188,206] or performing a serial dilution prior to filtration [207]. Additionally, sample pre-treatment with enzymes can improve sample filterability [135,206]. After filtration, the filter with the deposited bacteria is incubated in a nutritive broth or an agar plate to grow and enrich the bacterial population [205]. Alternatively, the bacterial cells on the filter can also be retrieved using the principles of elution [135,206]. Filter type, pore shape, and pore dimensions all contribute to the ability to elute microorganisms from the filter [206]. The harvested cells can then be combined with other Raman-compatible isolation strategies such as centrifugation for bacterial analysis. Overall, during filtration care must be taken during processing of the sample to cause minimal stress to the bacteria with respect to such factors as processing time, vacuum pressure, and desiccation [180] as they can impact the Raman spectra, and thus, affect the identification of bacterial species. In more sophisticated work, filtration has been combined with other methods to isolate bacteria from complex matrix in preparation for Raman measurements. For instance, Ravindranath et al. [208] combined nano-porous membranes with gold or silver nanoparticles labeled with antibodies for SERS spectroscopy to filter samples of bacteria in a buffered physiological solution. Bacteria bound to the antibodies remained in the membranes while unbound bacteria were filtered out. 

While filtration may be a commonly used method for isolation of bacteria, it has some drawbacks. As already highlighted, to ensure effective bacterial capture, the filter’s pore size must be sufficiently small, which can limit the types of filters and sample volumes that can be processed [209]. Additionally, microorganisms may adhere to the filter’s surface or become trapped within its pores, resulting in incomplete bacterial recovery [210]. As a result, filtration extracting bacteria from filters can be a complex undertaking.

**Figure 8:**
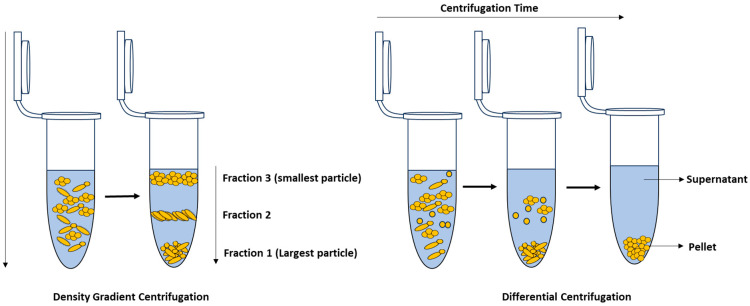
Density gradient centrifugation (**left**); differential centrifugation (**right**).

*Centrifugation:* Centrifugation is an isolation technique that separates particles or cells in a liquid medium by applying a rotational force around a fixed axis. This force propels particles or cells in a liquid medium to sediment, with the rate of sedimentation dependent upon a variety of physical parameters such as particle diameter, particle density, solution density, angle, and rotation speed [206]. Therefore, by modifying variables such as solution density and particle size, alternative centrifugation methods such as density gradient and differential centrifugation have been developed (Figure 8) [6].

Differential centrifugation exploits the principle that particles of different sizes or densities will settle at different rates, with the largest and most dense particles settling the fastest and leaving smaller and less dense particles in the supernatant [6,206]. By employing successive adjustments in centrifugation speed, the particles or cells with higher densities are separated from those with lower densities at each stage. The centrifugation speed is then increased until the target cells settle, after which the final supernatant is removed and the pellet is resuspended for further analysis [206]. A typical application of differential centrifugation is using low-speed centrifugation to remove heavier particles in food samples before applying high-speed centrifugation to sediment bacterial cells.

In contrast, density gradient centrifugation is based on separating the individual components of a complex sample according to their densities and sizes using a special gradient medium [105,211]. During centrifugation, the sample is spun through the gradient, and the individual particles then migrate to the portion of the gradient that is at equilibrium with its own density and form a “band” or layer, which can be extracted for further analysis [105,206]. Density gradient centrifugation can be employed for size and mass-based separation, known as rate-zonal centrifugation, or for separation based on density, often referred to as isopycnic or buoyant density centrifugation [211]. As such, this technique is widely employed for isolating bacteria from samples for Raman analysis as demonstrated in [105,135,186,212,213]. Materials commonly used to generate density gradients include sucrose, Ficoll^®^, OptiPrep^TM^, and Percoll^®^ [105,186,206,214]. Wichmann et al. [105] have developed a Raman-compatible technique that enables the isolation of bacteria from bronchoalveolar lavage (BAL) through density gradient centrifugation. To prepare the density gradient, OptiPrep^TM^ and a Galantine-buffered solution (GBS) were mixed in various ratios to achieve densities of 1.1066 g/mL, 1.0903 g/mL, 1.0631 g/mL, and 1.0576 g/mL, which were ideal for isolating bacteria. The gradient was then pipetted into a 5 mL Falcon tube, and a sample was added on top of the gradient, which was then centrifuged in a swinging-bucket rotor (2700× *g* for 60 min at 23 °C). After isolation, the pellet was washed three times with distilled water by centrifuging for 5 min at 10,000× *g* and 23 °C. The yield of the isolation method was evaluated using a pure culture of *S. thermophilus*, which resulted in a recovery rate ranging from 63 to 78%. To determine the impact of the gradient on bacterial spectra, a pure *S. thermophilus* without sputum was used. The results showed no differences between the mean spectra of bacteria isolated using the gradient and those from a pure culture.

Hence, one important consideration in this technique is the choice of gradient medium and its impact on effective separation and cell properties. It is essential that the gradient medium does not adversely affect cell properties; for instance, it should maintain physiological ionic strength to prevent cell lysis or dehydration effects. Moreover, the medium should be nontoxic and not compromise cell viability [206,215]. 

To prepare the samples for Raman measurements, the resulting pellet is typically resuspended in distilled water and the suspension is washed three times to clean it from residues from the medium which might interfere with the Raman spectra [105,135,186]. 

Centrifugation methods have achieved some success, offering the added advantages of easy processability and rapid handling; yet, they do have limitations. In some cases, a single centrifugation step may not suffice to effectively isolate bacteria from samples, necessitating multiple centrifugation steps, a common occurrence in cell preparation protocols [216]. For this reason, centrifugation is sometimes applied in conjunction with other methods such as filtration and enzymatic procedures.

Numerous studies have been conducted on the susceptibility of bacterial cells to centrifugation. Mixed outcomes have been reported, primarily due to the absence of a straightforward method for predicting or assessing bacterial cell damage caused by this process [216,217]. Thus, the impact of centrifugation on bacteria isolation remains a complex phenomenon shaped by a variety of factors, including the speed, duration, temperature, and type of rotor used for centrifugation, as well as the bacterial species, growth phase, and culture conditions [217,218,219]. Nonetheless, some studies have suggested that centrifugation has the potential to modify bacterial cell surface properties and internal structures, including DNA. These alterations can occur by stripping them off, compressing them, or even, in some extreme cases, cell blebbing as a consequence of the numerous shear stresses they are subjected to during the spinning process [216,217,218,219,220]. These may have a negative impact on bacterial identification by Raman spectroscopy. Garcia et al. [129] investigated the effect of multiple centrifugation steps, among other factors, on Raman spectra and discovered that excessive centrifugation introduces noise in Raman spectra, influencing accurate bacterial identification. In light of this, it is imperative to thoroughly assess the potential impact of centrifugation on bacterial cells during the sample processing stage and to carefully optimize the centrifugation parameters. This optimization is essential to guarantee minimal alterations to bacterial cells and, consequently, to prevent any interference with the Raman spectra obtained.

*Dielectrophoresis (DEP):* Dielectrophoresis is an electrokinetic phenomenon which exploits the effect of a non-uniform electric field on a particle in a three-dimensional space [6,189,221,222]. The DEP force depends on the particle volume, its relative polarizability, and the suspending medium, as well as the spatial change in the electric field [223,224,225,226]. Given that most biological cells behave as dielectrically polarized particles in external electric fields, they will experience a DEP force when subjected to alternating current (AC) or direct current (DC) non-uniform electric fields, which will drive them towards or away from the field gradient depending on their polarizability relative to the surrounding medium [215,227]. This selective movement enables the isolation and concentration of bacteria from complex samples, such as blood or urine, prior to Raman spectroscopy analysis. 

**Figure 9:**
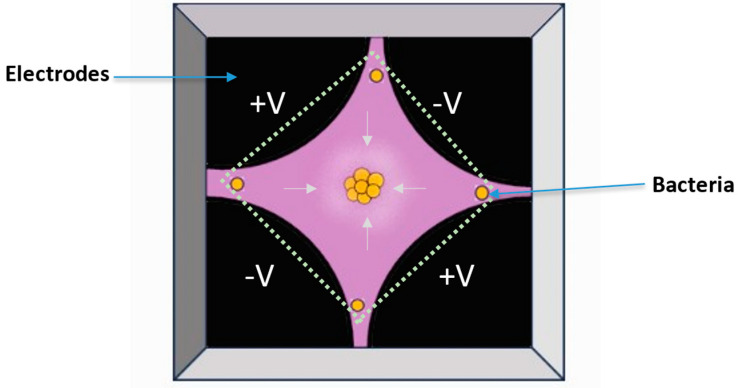
Layout of a Raman-compatible dielectrophoresis chip (adapted from [6], copyright 2015 Elsevier).

Advances in electrode-based DEP device design have resulted in the development of insulator-based and interdigitated microelectrode (IME)-based Raman-compatible DEP microchips or microfluidic devices (Figure 9) [189,228,229,230]. Typically, for DEP isolation and Raman measurement, a droplet of bacteria dispersed in a suitable liquid medium (a few hundred microliters) is injected or deposited on top of the Raman DEP chip. Next, a non-uniform electrical field for negative dielectrophoresis (nDEP) is generated by applying a suitable alternating voltage and frequency, and bacterial cells accumulate at the chip’s center, where their Raman spectra are recorded [189,228].

Using dielectrophoresis and Raman microspectroscopy, Schröder et al. [188] developed a method to identify and characterize individual bacteria from urine samples and to distinguish between *E. coli* and *E. faecalis*, two common causes of urinary tract infections. Here, a dielectrophoresis–Raman chip was used for analyzing urine samples from patients with single-pathogenic urinary tract infections, after filtering out larger particles such as leukocytes or epithelial cells. The proposed assay requires only 35 min for sample preparation and is suitable for diagnosing significant concentration of bacteria (10^5^ cells/mL). Hanson et al. [230] also have developed a contactless DEP–Raman device that combines dielectrophoresis and Raman spectroscopy for simultaneous isolation and label-free identification of bacteria. The device successfully isolated bacteria from a mixed sample consisting of *Mycobacterium* spp. and 3 μm polystyrene spheres and acquired Raman spectra of the trapped bacteria, demonstrating its potential to decrease the analysis time, particularly for diagnostic purposes. The spectra of the isolated bacteria were classified with an overall accuracy of 100%.

However, to achieve accurate isolation of bacteria for Raman spectroscopy, several key considerations must be taken into account. Primarily, the choice of the dielectrophoresis parameters such as the frequency, the amplitude of the applied electric field, and the time period in which the cells are exposed to the electric field are crucial [189,224]. These parameters should be optimized to ensure that the bacteria of interest are efficiently trapped and manipulated while minimizing damage or alteration to their biological properties as well as preserving cell viability. 

Also, the choice of electrode configuration or geometry as well as the composition plays a significant role in achieving accurate isolation [225,227]. Microfabricated electrodes with well-defined and highly symmetric geometries can provide precise control over the electric field gradient, improving the selectivity of bacterial trapping [189]. Metallic electrodes are prone to electrode fouling and electrolysis, producing hydrogen and oxygen gas bubbles that limit electrode operation, thus their effects need to be considered [230]. 

Furthermore, it is essential to carefully consider the suspension medium and its conductivity. A high conductivity of the surrounding medium can impact both the magnitude of the DEP force and the direction of the dielectrophoretic response of bacteria [221,231]. Joule heating also poses a risk, causing high temperatures that could harm biological cells and additionally introduce electro-thermal induced fluid flows that can interfere with or dominate the DEP force [189,230,231,232]. As highlighted several times, bacteria, like other single-cell organisms, respond to their environment and media and, as such, are sensitive to medium factors such as pH, conductivity, and electrolyte valency. Therefore, the influence of the surrounding media must be considered and kept consistent when planning DEP experiments [229].

Lastly, it is also important to ensure that the bacterial sample is free from contaminants or debris from the matrix that could interfere with Raman spectroscopy measurements. It is, therefore, advisable to include pre-filtration steps, followed by a medium exchange/washing step and resuspending the washed bacteria in an appropriate buffer/medium during the sample preparation step for measurements [189].

**Figure 10:**
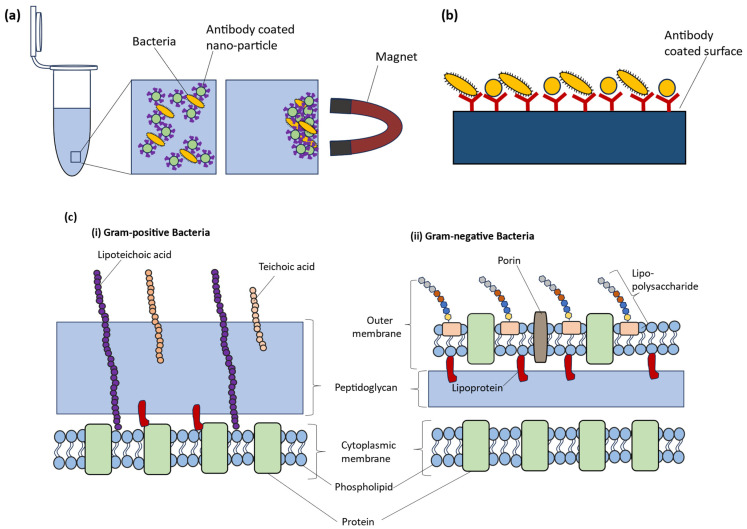
Schematic diagram for (**a**) immunomagnetic separation (**b**) capturing bacteria via antibodies (adapted from [6], copyright 2015 Elsevier) and (**c**) depiction of surface properties of (**i**) Gram-positive bacteria and (**ii**) Gram-negative bacteria.

*Immunoaffinity and targeting cell wall structures:* Immunoaffinity and targeting cell wall structures are two methods that can be used to isolate bacteria for Raman analysis (Figure 10). Immunoaffinity is a method that relies upon the specificity of monoclonal antibodies directed against specific and unique cell-surface antigens on the surface of bacteria [206]. Since the antibody–antigen interaction has a very high specificity, antibodies are ideal for capture and isolation of intact cells directly from complex sample suspensions [187,206]. Antibodies are proteins that recognize and attach to foreign molecules, such as bacterial cell wall components. In this technique, antibodies are affixed directly to a solid substrate, such as magnetic beads or nanoparticles, and subsequently introduced to a bacterial sample. The bacteria that adhere to the antibodies can be isolated from the sample through the application of a magnetic field [233] or centrifugation [157] (Figure 10). Following separation, the bacteria undergo Raman spectroscopy to acquire their unique spectral signatures. 

A novel method for detecting *S. aureus*, a common foodborne pathogen, was developed by Ji et al. [234], using functionalized magnetic beads and surface-enhanced Raman scattering (SERS) tags. Polyethylene glycol (PEG) and bovine serum albumin (BSA) dual-mediated teicoplanin-functionalized magnetic beads (TEI-BPBs) were used for the isolation of the target bacteria. TEI can recognize and capture Gram-positive bacteria, such as *S. aureus*, by binding to the _D_-Ala–_D_-Ala peptide fraction; however, it lacks some specificity in recognizing the bacterial cells [234]. Therefore, SERS tags were used to immobilize antibodies on gold surfaces via bifunctional linker proteins to ensure specific recognition of *S. aureus*. Samples of *S. aureus* were then incubated with both types of nanoparticles, which bind to the bacterial surface and allow magnetic trapping and SERS-based detection. Under ideal conditions, the combination of TEI-BPBs and SERS tags showed reliable performance, with high capture efficiency even in the presence of 10^6^ CFU/mL of non-target bacteria. The SERS tag provided an effective hot spot for subsequent Raman detection, with a detection limit of 1 CFU/mL and a 10^2^–10^7^ CFU/mL detection range. The method also performed well when tested on milk samples, with a high recovery rate of 95.5–101.3 ± 2–3%.

Although this approach has some advantages, its success depends on several factors: the specificity of the antibody used, the dimensions and surface characteristics of the antibody-coated particles, the efficiency of the recovery process, and the potential interference from the sample matrix [235,236,237]. On the other hand, a major drawback of traditional immunosensor assays is the high specificity of antibodies, which necessitates multiple antibodies when screening for various targets within a sample. Moreover, it can be challenging to obtain pathogen-specific antibodies, since often antibodies are specific only at a genus level, attributed to the presence of shared cell-surface structures such as proteins or carbohydrates [187,238,239].

Complementing the utilization of antibodies specific to bacterial cell wall constituents, an alternative strategy involves a broader approach using immunoglobulins that target functional surface structures or characteristics found in both Gram-positive and Gram-negative bacteria (Figure 10) [187]. Both Gram-positive and Gram-negative bacteria have a cell membrane and a sacculus made up of peptidoglycan. In contrast, in Gram-negative bacteria there is an additional outer membrane, which is a phospholipid bilayer containing lipopolysaccharides. Therefore, in the presence of Gram-negative bacteria, antibodies will specifically target lipopolysaccharides, whereas in the case of Gram-positive bacteria, they will target the cell wall’s lipoteichoic acids [187]. An application of this approach was demonstrated by Pahlow et al. [187], who used a chip-based layout for the isolation of various bacteria with different cell wall structures from a buffer, employing antibody-mediated capture. The detection limit for this technique is in the region of 10^4^ cells/mL in buffer solution. Additionally, the components of the bacterial cell wall can impact the way the cell interacts with its surrounding environment. This, in turn, can affect the cell wall’s charge, hydrophobicity, and rigidity. These factors can be utilized as a basis for bacterial isolation. For instance, certain techniques use cationic polymers, hydrophobic ligands, or enzymes to bind specific cell wall components and isolate bacteria based on their affinity [206]. Mircescu et al. [240] demonstrated how bacteria can be immobilized through electrostatic forces by using chemically modified glass slides instead of specific capture molecules. Silanization and diamino-PEG-ylation were used to introduce terminal-amine-groups on the glass slides. The slides were then treated with HCl to protonate the amine groups, resulting in a permanent positive charge on the surface. This positive charge was used to capture *E. coli* cells, which have a negative charge on their cell walls due to the presence of the phosphate and carboxylate groups of lipopolysaccharides. These groups are typical for Gram-negative microorganisms. The advantage of this method lies in its broad-spectrum applicability, negating the requirement for specialized capture probes tailored to individual bacterial species.

**Figure 11:**
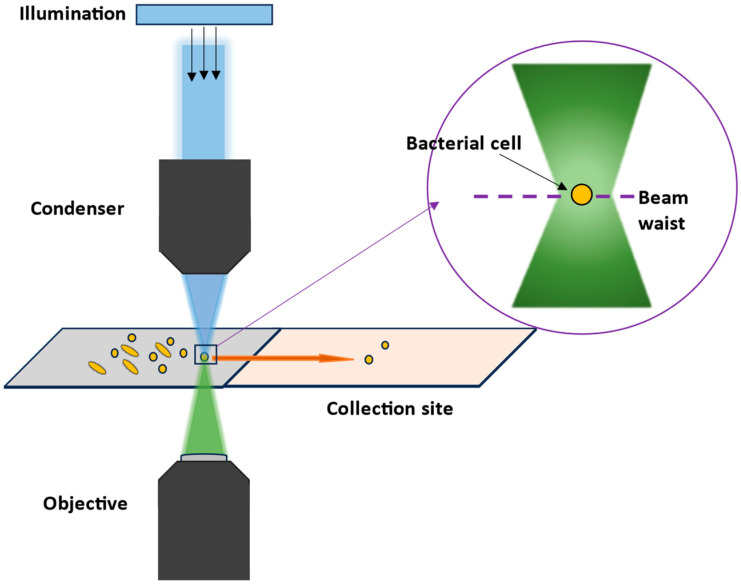
Schematic diagram for an optical trap system for sorting bacteria cells.

*Optical tweezers*: Optical tweezers, also known as optical traps, employ a highly focused laser beam for contactless micromanipulation, immobilization, and precise resolution of individual bacterium cells suspended in a solution [116,241,242,243,244,245,246,247,248]. The formation of optical tweezers involves the generation of such a beam, achieved by using a high-quality objective lens, to establish a focal point in the specimen plane (Figure 11). This focal point creates an optical trap capable of retaining small particles at its center. If a cell is nearby, it interacts with the laser and, due to the transfer of momentum from the incident photons, is drawn into the trap by gradient optical forces. The trapped cell may be moved in three dimensions by either moving the focal point or translating the sample chamber while maintaining the trap fixed [243,249,250,251].

Therefore, this method is an effective tool for the sorting of cells, especially as a strategy for the selective isolation of microbial cells from a diverse population [6,249,250]. When combined with Raman spectroscopy, this approach is useful for investigating microbial communities and identifying cells of interest, which is valuable for studying unculturable and unknown species [244]. Additionally, optical tweezers allow levitation of cells above the substrate, which minimizes fluorescence effects and interference from other cells in the measurement while keeping a particle in an optical trap, providing the best possible excitation and collection of Raman spectra [242,250,252].

A decade ago, Huang et al. [253] developed a dual-laser optical tweezer system for measuring and sorting single yeast and bacterial cells according to their Raman spectra. The laser system included a 514.5 nm laser to perform Raman measurements and a 1064 nm laser to manipulate the cells. In this study, a combination of yeast cells (*Saccharomyces cerevisiae*) and two bacterial species (*E. coli* and *P. fluorescens*) was employed, and the sample comprised 10^5^ cells/mL of each species in equal quantities. Cultivation successfully recovered over 50% of the isolated yeast cells and over 40% of the *P. fluorescens* cells. Genome amplification of individual cells was undertaken to demonstrate the suitability of this technique for investigating unculturable bacteria. Two out of seven sorted yeast cells and three out of eight sorted bacterial cells were genome-amplified correctly. More recently, Lee et al. [254] have also developed an innovative automated sorting platform that combined Raman spectroscopy, optical tweezers, and microfluidics to sort individual cells based on their physiological traits or functionalities of interest. This method has several advantages over manual sorting, including higher throughput (up to 500 cells per hour) and accuracy (98.3 ± 1.7%). It is also fully automated, eliminating any user biases that could arise with manual sorting. The entire process takes around 4 h, depending on the number of cells needed, and involves a 1-day preparation of cells.

All the same, several aspects must be considered before optical tweezers may be used for microbial studies. The laser parameters, which include wavelength, laser beam quality, and laser power, are the most important factors that may impact sample viability, trapping efficiency, and the ultimate signal-to-noise ratio (S/N) during measurements [243,250,255]. The quality of the laser beam is crucial in generating the tightly focused spot necessary for optical trapping, which should be as close to the diffraction limit as possible. Good pointing stability is also vital to keep the optical trap’s position steady. In optical trapping studies, changes in laser power can cause variations in the strength of the optical trap. Therefore, it is essential to monitor variations in laser power, which are typically measured by two indicators: noise (i.e., variations around the average laser power within a given bandwidth) and power stability (i.e., the drift in the average laser power measured over an extended period) [255]. However, as several studies have shown, single cells can be very sensitive toward laser illumination. And while increasing the laser power can enhance the optical forces that capture and manipulate bacteria, it can also cause more heating, leading to alterations in the biochemical composition and structure of the cells due to photodamage or photochemical reactions [250,253,256,257]. This might ultimately influence the Raman spectra of the trapped cells. Thus, the optimal laser power should strike a balance between cell viability and trapping stability. Other internal parameters, such as the refractive index of the sample medium as well as the bacteria’s refractive index, size, shape, and position, may also have a direct influence on the optically stable trap and must be considered [258]. Finally, before obtaining the Raman spectra, cells must be washed with distilled water to reduce the influence of the growth media and any matrix interference.

## 6. Statistical Evaluation and Data Modeling

Data analysis is crucial for realizing the full potential of Raman spectroscopy. It converts raw spectral data into useful information to achieve aims including quantitative analysis, pattern recognition, and data interpretation, in addition to accurate molecular identification. Raman spectroscopic analysis protocols and pipelines are explained comprehensively in references [259,260,261,262]. 

**Figure 12:**
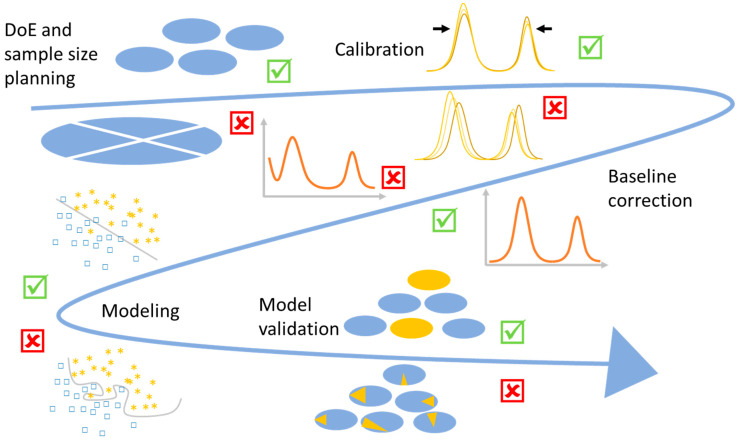
A visualization of Raman data analysis pipeline and the possible mistakes. The mistakes may arise from the sample size planning, which should determine enough independent observations. Subsequently, calibration is performed for both wavenumber and intensity. While baseline correction is crucial, it should be followed by data normalization. The model’s complexity is directly associated with the quantity of independent observations, with an emphasis on avoiding excessive complexity. Thereafter, an unbiased estimation of the model generalizability is needed, which requires validation over independent measurements.

The data analysis pipeline for Raman spectra is a series of steps to ensure the accuracy and reliability of the information extracted from the measured Raman data (Figure 12). This data pipeline for Raman spectra contains a sequence of algorithms, each serving a specific purpose in enhancing the quality and interpretability of the data. However, it is crucial to recognize that errors can occur when applying these algorithms, and it is important to avoid these errors.

In the following, methods of experimental design, data pre-treatment and pre-processing, and data modeling are introduced, along with a review of the possible mistakes. 

### 6.1. Design of Experiment

When conducting a biological study using Raman spectroscopy, research objectives and analytical goals (exploratory, diagnostics, biomarker identification, …), sample limitations (e.g., impurities), and the suitable Raman instrument should be considered. Therefore, the design of experiments (DoE) is crucial for successful studies to obtain clear answers to key questions about the task and sample properties. Also, it is used for determination of the minimum number of samples that is needed to capture the essential part of a population. DoE comprises two main parts: the measurement protocol and the sample size planning (SSP). Thereby, the measurement protocol contains determination of the spectrometer, sample preparation, and the spectrometer calibration procedure [263,264]. 

A good measurement protocol helps to reduce the impact of factors such as instrumental factors on the Raman spectra. One common mistake is overlooking the appropriate laser power and exposure time. Using a high laser power may cause sample degradation or unwanted heating effects, while insufficient exposure time may lead to weak signal intensity and poor signal-to-noise ratio. It is important to optimize these parameters based on the sample characteristics and the desired level of sensitivity and resolution.

Sample size planning determines the minimum number of samples needed for reliable conclusions or for model building with satisfactory performance. One of the initial and crucial considerations in research is planning the appropriate sample size. SSP can be determined by examining the learning curve that represents a model performance measure like accuracy as a function of the sample size. The goal is to identify the minimal sample size at which the performance measure no longer exhibits significant improvement [265,266,267]. It is typical to determine the sample size on the spectra level, but also on the highest level in the sampling hierarchy, like biological replicates and patients. 

The number of samples is particularly important when working with data-driven artificial intelligence (AI) models. To ensure the reliability of these models, it is recommended to have a minimum of three independent replicates for studies, although having five is even better as outliers can be identified. This enhances the robustness of model training and testing. In the context of diagnostic studies, where accuracy is paramount, a more substantial patient sample size is advised, typically ranging from 20 to 100 patients [265,268]. This larger group of patients is especially critical when it comes to evaluating the model’s performance. A sufficient sample size in diagnostic studies strengthens the statistical reliability of the results.

### 6.2. Pre-Treatment Methods

Raman spectroscopic data analysis begins with several pre-treatment steps. Within this context, spike removal and calibration are discussed as they play pivotal roles in enhancing data quality and robustness.

*Spike removal*: Cosmic spikes are narrow and intense peaks that can randomly appear in Raman spectra due to the high-energy cosmic particles that hit the CCD detectors [269,270]. These intense peaks make data analysis difficult. Normalization and feature extraction may yield non-meaningful outcomes without spike removal. Spikes should be removed at the first step of data analysis because some later pre-processing methods like interpolation broaden the spikes and make it difficult to distinguish between spikes and Raman peaks.

Specialized algorithms are used to remove spikes in Raman spectra; they remove the spikes but preserve sharp peaks. Spike correction algorithms identify and eliminate spikes and a number of methods like mean comparison, Laplacian operators [150], wavelet transforms [271,272], or modified Z-scores [273] can be used for this task. First, a threshold is applied to the calculated metric to detect cosmic spikes. In reference [150], an automatic detection method is employed, using a threshold on the normalized Laplacian response. In contrast, the authors of reference [274] detected spikes visually. After being detected, a spike can be removed by linear interpolation based on the two boundary points of the spike or by other interpolation methods, e.g., spline interpolation.

*Calibration*: In Raman spectroscopy, it is important that the recorded spectra are consistent across different instruments, experimental conditions, and over time. However, variations which are unrelated to the sample often occur, and to overcome this spectrometer calibration, including wavenumber and intensity calibration, is used. These calibration methods are used to standardize spectra and remove unwanted changes, ensuring consistency and reliability. 

In practice, it is recommended to perform wavenumber calibration using a substance like 4-acetamidophenol, which exhibits numerous distinct peaks within the region of interest. These measurements serve as the foundation for creating a tailored wavenumber axis for each measurement day. Subsequently, these wavenumber axes are harmonized through interpolation to establish a consistent and clearly defined wavenumber axis [275,276]. This calibration is necessary as it prevents significant systematic drifts that may arise in the measurement system, which could otherwise obscure the interpretation of sample-related changes. Therefore, wavenumber calibration should not be skipped.

After the wavenumber calibration, the Raman spectra are subjected to an intensity calibration. Intensity calibration is performed using a known standard material with known Raman intensities across the desired wavenumber/wavelength range. To perform intensity calibration, the intensity response function is calculated by comparing the observed intensity with the expected intensity (from a calibrated spectrometer) of the standard material. One version for correction of the intensity values in a Raman spectrum is the subtraction of the measured dark current spectrum from the measured Raman spectrum and then the result is divided by the calculated intensity response function. This process provides more accurate intensity values for analysis [277,278].

The optimal spectrometer calibration remains a subject of ongoing research, but certain steps can be taken. For instance, it is advisable to conduct standard measurements at regular intervals, such as daily, weekly, or whenever modifications are made to the setup. These measurements, whether they involve the wavenumber standard or white light spectra, serve a dual purpose: they support the calibration process and function as quality control checks, ensuring the reliability of the data.

### 6.3. Pre-Processing Methods

Pre-processing involves the application of specific methods to eliminate undesired contributions or disturbances that originate from the instrument or the sample. Its primary purpose is to refine the measured data by removing these unwanted effects, thereby isolating and enhancing the pure Raman signal of interest, facilitating more accurate and meaningful analysis [279,280,281,282]. This pre-processing workflow typically begins with a baseline correction, as the Raman spectra often share the same energy range as fluorescence, causing an overlap. Common techniques for correcting the fluorescence background of Raman spectra are the sensitive nonlinear iterative peak (SNIP) clipping method [283] and asymmetric least squares smoothing [284].

Subsequently, denoising or smoothing methods [285] like Savitzky–Golay, Gaussian, median, or moving average smoothing are applied to reduce unwanted noise. In the next step, a normalization [286] is executed to ensure that spectra obtained on different measurement days and conditions can be effectively compared and analyzed. Normalization methods like vector normalization, standard normal variate (SNV), maximum normalization, peak area normalization, and min–max scaling are used.

It is crucial not to introduce excessive data manipulation, known as over-processing, during the pre-processing. Over-processing involves removing data components that may carry valuable information about the sample rather than just eliminating sources of variance like instrumental or environmental variations. For example, over-smoothing can remove a shoulder peak from a larger peak and may degrade the subsequent analysis. Such a process is recommended only for highly noisy data. Improper baseline correction parameters can make peak maxima shift away significantly from their original position and multiple peaks can be fit to a single peak; however, this may not be based on reality. 

Choosing the best method and parameters for pre-processing in Raman spectroscopy is challenging [287,288]. Therefore, no universal solution exists, and automatically selecting the parameters can be difficult. One common approach is to optimize a parameter based on the results of a subsequent analysis, such as a regression or classification model. However, that must be performed with extreme caution to avoid overfitting. It is desirable to use spectral markers as the merit of such optimization rather than the model’s performance.

In addition, the normalization should be applied after baseline correction in a pre-processing procedure. The fluorescence background can be 2–3 orders more intense than the Raman bands and applying normalization before background correction results in a strong bias of the normalized Raman spectra. This issue should be avoided.

### 6.4. Modeling

Once the Raman spectra have been pre-treated and pre-processed, statistical or machine learning models are employed to extract information of interest, such as substance concentrations, substance distribution, or sample classification. Dimension reduction, model construction, and model evaluation are three important parts of data modeling.

Dimension reduction serves the purpose of extracting valuable features from the data while eliminating redundant information and suppressing noise. Dimension reduction methods contain unsupervised methods like principal component analysis (PCA) and supervised methods like partial least squares (PLS) regression [289,290]. The number of principal components (PCs) and latent variables (LVs) in PLS are important and need to be selected wisely. If too few variables are included, trends in the dataset may not be sufficiently characterized. A higher number of variables often contain more noise, so this should be avoided. The number of vectors must be optimized to minimize computation time while maximizing prediction accuracy [291].

The output of dimension reduction is fed into a statistical or machine learning model, translating spectral data into meaningful information. Both models are built using a portion of the dataset known as the training or classification data. The model’s performance is then assessed and evaluated using the remaining portion, referred to as the testing or validation data. This separation allows us to understand how well the model generalizes to new, unseen data. 

The choice of the model and its complexity should be tailored according to the number of independent measurements available. For extensive independent datasets, it is possible to employ highly parameterized models like deep learning models [292,293,294,295]. Conversely, when dealing with limited independent observations, it is preferable to use simpler models such as linear models [296,297,298].

Convolutional neural networks (CNNs) and support vector machines (SVMs) have received widespread attention in Raman spectra analysis. For instance, a CNN was utilized to rapidly identify *Salmonella* serovars (70/30% training/test split) with a prediction accuracy of over 98.5% [299], to distinguish between live and dead *Salmonella* with 20-fold cross-validation and an average accuracy of 98.7% [300], and to discriminate clinically significant pathogens with 99.9% accuracy with samples were divided into training set, validation set and test set by following the ratio of 6:2:2 [301]. Raman spectroscopy combined with SVM was used to identify 21 microorganisms [302]. The validation set, which was not included in the calibration dataset, had a prediction accuracy of approximately 80.0%.

Zhang et al. used eight powerful machine learning algorithms to determine the best classifier for discriminating between periodontal pathogens, namely, *Porphyromonas gingivalis* (*Pg*), *Fusobacterium nucleatum* (*Fn*), and *Aggregatibacter actinomycetemcomitans* (*Aa*), analyzed by Raman spectroscopy. The study can be found in [303]. The algorithms used in this study include extra trees, AdaBoost, gradient boosting, linear discriminant analysis, support vector machine, multi-layer perceptron, passive-aggressive classifier, and quadratic discriminant analysis. The data were randomly divided into training (75%) and testing (25%) sets at the sample level. Each sample was measured repeatedly, producing 10 spectra. The classifiers’ performance was evaluated using various metrics. They included the receiver operating characteristic (ROC) curve, the area under the ROC curve (AUC), accuracy, confusion matrix, sensitivity, specificity, and F1 score. The machine learning algorithm extra trees achieved the highest accuracy of 94.7% at the sample level and 93.9% at the spectrum level in the three-class discrimination models. 

Sil et al. distinguished between 15 DNA samples extracted from the *Brucella* and *Bacillus* genera [304]. The Raman spectra of these DNA samples exhibited unique features that could be attributed to specific markers. The study found that *Bacillus anthracis* has unique Raman DNA signatures that distinguish it from *Bacillus cereus* and *Bacillus thuringiensis*. This differentiation was achieved by principal component analysis (PCA), hierarchical cluster analysis (HCA), and principal component analysis-linear discriminant analysis (PCA-LDA). The feasibility of clustering the different DNA samples was investigated by using unsupervised methods such as PCA and HCA. A supervised algorithm, PCA-LDA, was used for classification. The PCA score plot showed that the *Brucella* species clustered away from the *Bacillus* DNA. As the Raman spectra reflect the biochemistry of the measured DNA samples, the distance between the two samples in the HCA dendrogram is a revelation of the biochemical dissimilarity of the different spectra. HCA was able to correctly cluster the DNA samples using an initial guess of 15 clusters given to the software. The overall accuracy of 86% was obtained in a 20-fold cross-validation of the PCA-LDA analysis. Additionally, a convolutional neural network (CNN) architecture was employed to achieve 100% accuracy in discriminating all 15 DNA samples in a training/test split procedure [304].

Rodriguez et al. reviewed different machine learning methods used in bacteria analysis by Raman spectroscopy [16].

The prediction performance of the model should be evaluated after model construction. A common mistake in model evaluation is related to the data independence within the validation process. It is important that the datasets used for validation purposes remain independent of those used for training. To meet this critical requirement, it is crucial to ensure that biological replicates or patients, which serve as independent measurements, are exclusively assigned to either the training, validation, or identification data subsets.

Failure to adhere to this requirement can lead to a significant overestimation of the model’s performance. In such cases, the model may appear to perform exceptionally well during validation, which does not reflect its actual generalization capabilities to unseen, independent identification samples. Therefore, maintaining the independence of data subsets is a fundamental principle in robust model evaluation in Raman data analysis.

## 7. Summary and Outlook

Raman spectroscopy, with its various techniques, enables a wide range of applications for microbiological investigations, especially for the identification of bacteria (Table 1). Depending on the requirements and the degree of discrimination, the method must be adapted to the specific problem. For example, a combination of isolation and single-cell measurements can be used for rapid, cultivation-free screening of bacteria. For further measurements, a (short) cultivation is necessary to obtain sufficient biomass for the analyses of bulk measurements. In addition, an adapted statistical evaluation with a suitable spectrum pre-treatment is necessary to avoid overfitting the dataset.

By considering all the aspects of this review, the robustness of the achieved database as well as the reliability of the results will be dramatically enhanced. Such databases can reveal the high sensitivity of Raman spectroscopy towards high-performing identification routines for different sample types. In the end, these approaches are helpful in establishing methods which can be used for routine measurements in non-specialized spectroscopical laboratories. However, before this can be the case several challenges remain that are linked to the variety of possible fields of application as well as the required tools that need to be developed in order to make Raman spectroscopy routinely applicable. It is important to specify the application and incorporate all of its special requirements into the design of Raman-based tests. In many cases, larger databases need to be created, much larger than those existing for research purposes or proof-of-concept studies. Also, specific AI-based tools need to be designed to fulfil the required high-accuracy standards present in routine analysis and overcome possible robustness issues in the data that are caused by inter- or intrasample variations as well as other independent factors. Finally, the operators need to be trained to correctly evaluate Raman data and overcome the mindset caused by the label-dependent analytical methods that are currently the gold standard in almost all fields. 

The main merits of Raman spectroscopy of bacteria are the high speed and sensitivity that can be provided by label-free evaluation. On the other hand, the spectral signature is prone to be influenced by factors related to the sample workflow and handling. This disadvantage necessitates careful design of SOPs as well as robust and constant quality control to avoid complications. When these demanding standards are applied, however, the advantages of this analytical tool can be fully exploited. 

## Figures and Tables

**Table 1:** Summarizing the challenges of different Raman spectroscopic methods in respect to their excitation wavelength.

Excitation Wavelengths	Application	Advantages	Disadvantages
**UV** (244 nm or 257 nm)	Bulk analysis	- UVRR enhances nucleic acid and aromatic amino acid signals - UVRR has a higher S/N ratio	- Large amounts of biomass required - (Long) cultivation times - Photodegradation through UV excitation
**VIS** (mainly 532 nm and 633 nm)	Single-cell analysis	- Very good spatial resolution < 1 µm - Non-destructive analysis of individual cells - Provides information of the complete cell content - Analysis of heterogeneous samples possible	- Sensitivity to autofluorescence in certain samples - Requires suitable isolation method(s) - Resonance enhancement of non-important biomolecules (e.g., cytochrome, carotenoids, …)
**NIR** (785 nm or 1064 nm)	Bulk analysis	- Minimizes fluorescence - Provides information of complete cell content - NIR does not damage sample	- NIR produces lower-intensity bands - Large amounts of biomass required
